# Resilient Artificial Intelligence in Health: Synthesis and Research Agenda Toward Next-Generation Trustworthy Clinical Decision Support

**DOI:** 10.2196/50295

**Published:** 2024-06-28

**Authors:** Carlos Sáez, Pablo Ferri, Juan M García-Gómez

**Affiliations:** 1 Biomedical Data Science Lab Instituto Universitario de Tecnologías de la Información y Comunicaciones Universitat Politècnica de València Valencia Spain

**Keywords:** artificial intelligence, clinical decision support, resilience, clinical medicine, machine learning, data quality, fairness, trustworthy AI, regulation, AI regulation, AI Act, EHDS, European Health Data Space, emergency medical dispatch, clinical decision support systems

## Abstract

Artificial intelligence (AI)–based clinical decision support systems are gaining momentum by relying on a greater volume and variety of secondary use data. However, the uncertainty, variability, and biases in real-world data environments still pose significant challenges to the development of health AI, its routine clinical use, and its regulatory frameworks. Health AI should be resilient against real-world environments throughout its lifecycle, including the training and prediction phases and maintenance during production, and health AI regulations should evolve accordingly. Data quality issues, variability over time or across sites, information uncertainty, human-computer interaction, and fundamental rights assurance are among the most relevant challenges. If health AI is not designed resiliently with regard to these real-world data effects, potentially biased data-driven medical decisions can risk the safety and fundamental rights of millions of people. In this viewpoint, we review the challenges, requirements, and methods for resilient AI in health and provide a research framework to improve the trustworthiness of next-generation AI-based clinical decision support.

## Introduction

The advent of electronic health record (EHR) data sharing posed expectations for improved, trustworthy development of artificial intelligence (AI) in health through a larger volume and variety of data. However, the development process of health AI and the generalization and fairness of the resulting models face significant challenges due to the inherent biases, uncertainty, variability, and quality levels of real-world data (RWD). These challenges include variable information across different settings and over time, biases affecting underrepresented groups, uncertainty from lacking or overlapping information, or data quality (DQ) issues such as incomplete or implausible information. These issues can be present in training data feeding the AI learning or manifest de novo in the AI routine clinical use (Textbox 1). If health AI is not designed resiliently with regard to these RWD effects, potentially biased data-driven medical decisions can risk the safety and fundamental rights of millions of people.

**Clinical case with potentially suboptimal clinical decision support outcomes in a high-risk artificial intelligence (AI) system according to the European Union (EU) AI Act—Article 6(2)—Annex III.**

A medical emergency dispatch center receives a call from a professor informing about an aged 20 years female student showing apparent respiratory distress. After data input, with no known chronic respiratory disease reported, an AI triage support system confirms that the case is not life-threatening. They send a basic life support ambulance. Eventually, the patient died during transport. The autopsy revealed a pulmonary embolism: she had recently started taking oral contraceptives. Clearly, this lack of information affected the AI outcome. Should AI have reacted by warning about potentially high uncertainty or asked for more information?

Alternatively, without previous embolism, a similar case might have occurred in March 2020 as a then-unknown effect of the SARS-CoV-2 infection. Instead of using a static AI decision support system trained on prepandemic data, should AI have automatically adapted to the very recent data patterns to provide better outcomes on new data?

Many recent papers in the highest-impact medical journals warned about the increasing obstacles imposed by RWD challenges for health AI. Cabitza et al [1] warned about the effects of intrinsic uncertainty in medical observations and interpretations on the reliability and accuracy of machine learning (ML). They claimed to develop and validate ML adaptable to the variable nature and actual DQ of medical information. Chen and Asch [2] argued that just relying on past data can have diminishing effects on AI’s usefulness and future generalization, as well as the “butterfly effect” of tiny current variations into the future. Gianfrancesco et al [3] outlined the potential contribution to socioeconomic health disparities of ML based on biased data. Rajkomar et al [4] classified the availability of high-quality data and learning from undesirable past practices among the key challenges for medical ML, especially in RWD and nonimaging data from EHR. Google Health and DeepMind teams identified data variability as among the key challenges in delivering clinical impact with AI [5]. Besides, the COVID-19 pandemic highlighted potential limitations in medical AI from inadequate training and evaluation design and DQ and variability issues [6,7]. Not surprisingly, the European Commission has recognized DQ as a risk for the safety and fundamental rights assurance (FRA) in AI and included it along with other RWD challenges within the recently approved AI regulation (Article 10) [8,9] and among the significant issues for the quality and economy of the European Health Data Space (EHDS) [10-13].

We focus on ML as the methodological driver for current well-established health AI and clinical decision support systems (CDSSs) across diverse clinical fields. In health ML, new knowledge is learned computationally from data in a training phase, generally from dedicated data cohorts or the EHR. This knowledge is then used to assist decision-making for new cases in a prediction phase. At prediction, a CDSS can retrieve its inputs from the EHR or manual input. Potential data-related uncertainties, variability, and biases for health AI can arise both at the training and prediction phases (eg, see Table S1 in Multimedia Appendix 1 for some examples).

The definitions we provide in this work apply to multiple traditional and state-of-the-art ML approaches for CDSS, including predictive analytics and the potential upcoming use of conversational AI [14]. The related techniques include, but are not limited to, deep learning, ensemble models, and statistical methods, with knowledge acquisition schemes based on learning from scratch and using pretrained or foundation models [15,16].

## Resilient AI: Definition and Requirements

### Definition

We define resilient AI (RAI) as an AI that can automatically or semiautomatically adapt and react to unprecedented, uncertain, or undesired situations in real-world environments during model training and its use.

**Figure 1:**
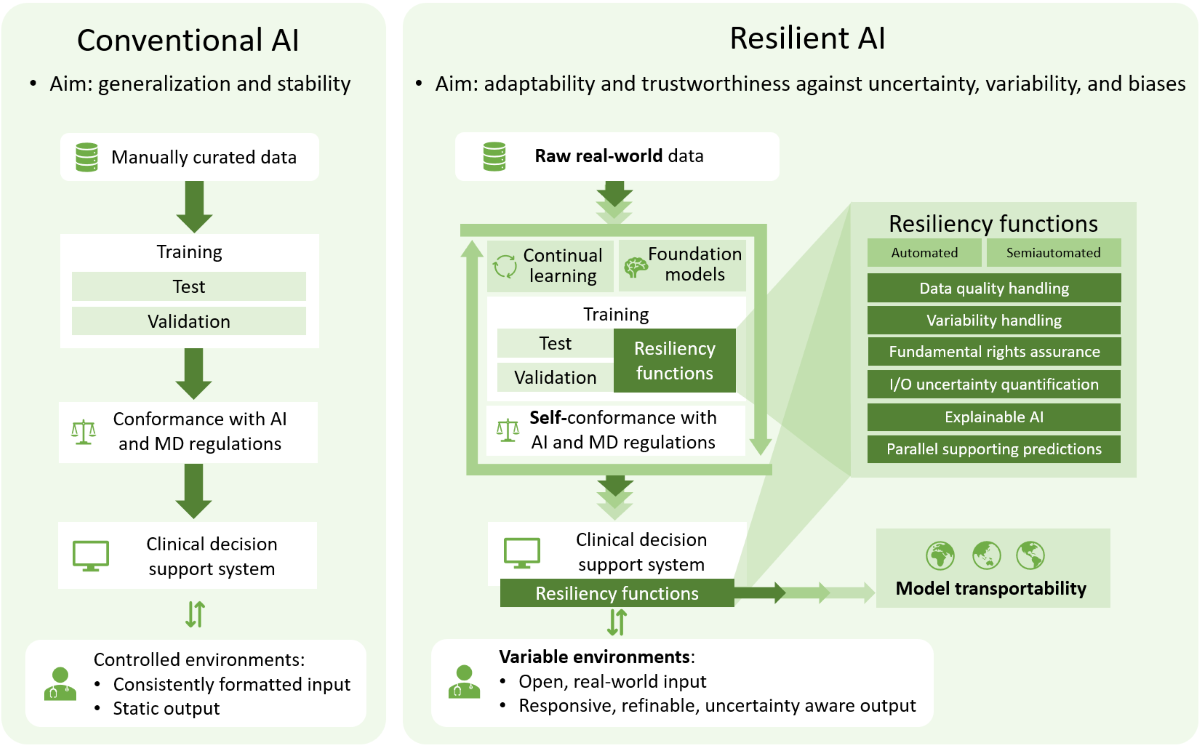
Comparison of the conventional AI process and the proposed resilient AI paradigm. Conventional AI is generally aimed at generalization and stability in controlled environments. That is, typically learning and validating a static model from a standardized, quality-controlled dataset, and applying the inference on equivalently consistent inputs. Resilient AI, however, aims to handle real-world variable environments during all its lifecycle, from adapting and explaining against potential biases and variability in the secondary use of electronic health records, both at model training and during use, to handling uncertainty in decision-making, enabling next-generation, trustworthy clinical decision support systems. AI: artificial intelligence, MD: medical device, I/O: input/output.

RAI emerges as a new paradigm over conventional AI procedures, generally aimed at model generalization and stability in controlled environments. The conventional AI approach expects learning on preprocessed, curated datasets and then inferring on equivalently consistent input data. The RAI approach, however, aims to learn and predict raw RWD by relying on resiliency-enabling methodologies and functions while improving its generalization and stability in variable environments. In RAI, adaptation would involve using new information to change or update AI knowledge, and reaction would involve providing appropriate information to support decision-making. Overall, RAI aims to enable trustworthy CDSSs for real-world environments, from adapting and explaining against potential biases and variability in the secondary use of EHR to handling uncertainty in decision-making, covering the whole AI lifecycle (Figure 1).

**Figure 2:**
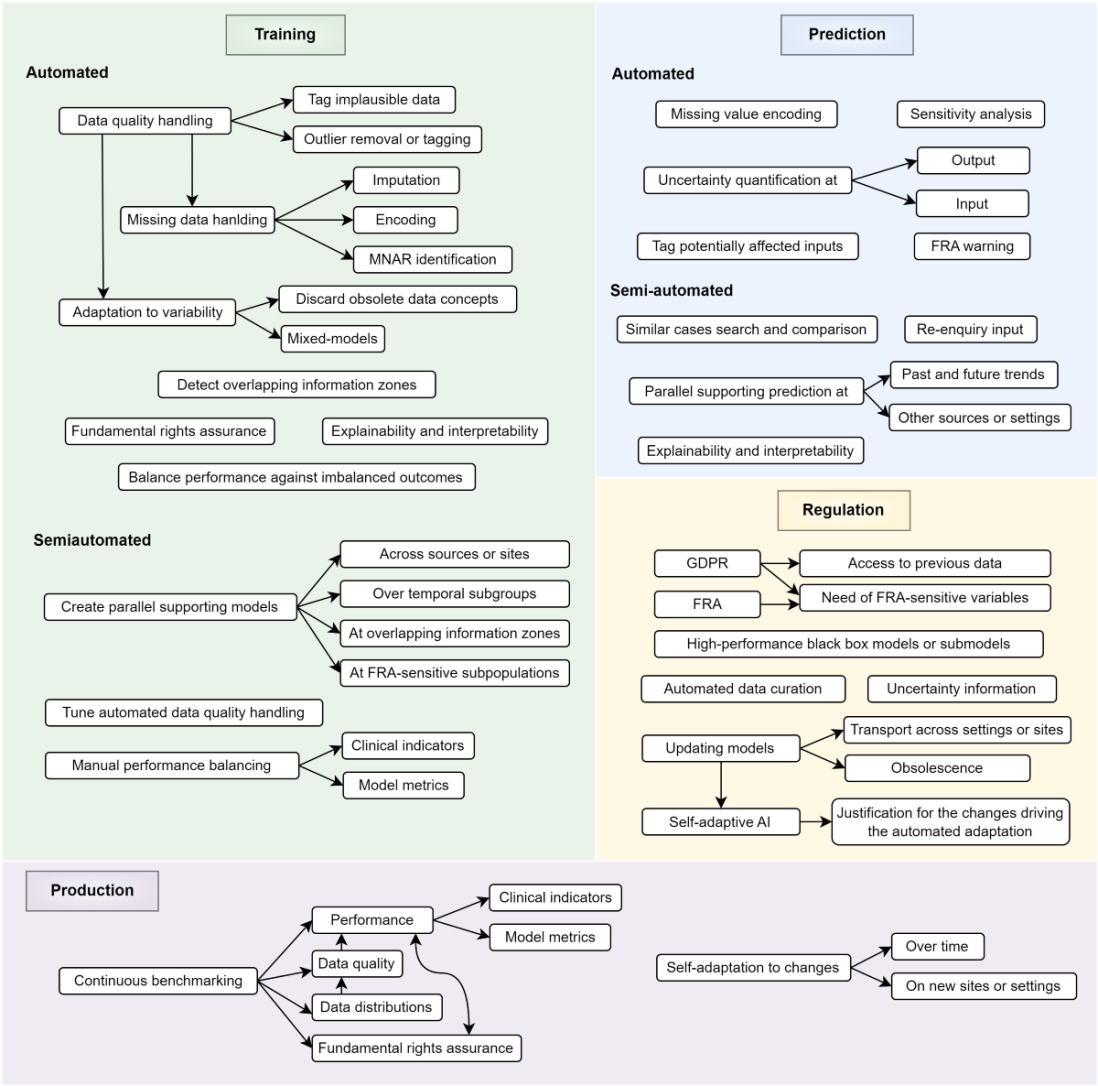
A classification for the expected behavior and functionality of resilient AI in health, regarding the AI training phase, the prediction in new cases, the routine maintenance of AI during production, and related regulatory aspects. We focus on supervised ML as the methodological driver for current well-established medical AI and clinical decision support systems. AI: artificial intelligence; FRA: fundamental rights assurance; GDPR: General Data Protection Regulation; ML: machine learning; MNAR: missing not-at-random.

The challenges for RAI include DQ issues, data variability over time or across sources, information uncertainty, human-computer interaction, security and privacy, and FRA, among others. By wrapping the desired behavior of health AI against these challenges, we propose a set of requirements for the behavior and functionality of RAI in health (Figure 2). These requirements are organized in the 4 blocks described next.

### Training

RAI should ensure quality, generalizable knowledge through automated or semiautomated data preparation and lifelong learning processes cost-effectively. Manual data preparation generally consumes 80% of the costs of AI projects, and many data issues neither generally addressed nor detected in apparently error-free data can potentially lead to biased or inaccurate models.

Most data-related barriers in training are categorized as DQ issues [17-19], including missing, implausible, or outlier data. RAI should tag DQ issues and allow models to discard or fix them, infer any useful knowledge associated with faulty inputs, or suggest multiple modelling pipelines. For example, repeated missing laboratory tests at emergency admission in patients with COVID-19 due to rapid intensive care unit admissions might lead to a missing not-at-random situation associated with higher severity in the outcome.

RAI training should be resilient against variability over time and across settings or sites. Changing or unexpected information patterns can present as shifts in the covariates—*p(x)—*prior probability shifts in the outcomes—*p(y)*—or as concept shifts in one conditioned to the other—*p(y|x) or p(x|y)*—leading to dataset shifts [20-22]. For example, would prediction models for acute respiratory distress syndrome trained on the first COVID-19 wave EHRs perform on new cases as initially evaluated? Would a model trained on a global European sample perform equally cross-border? These shifts can even relate to FRA-sensitive variables such as gender or race [10]. To delineate and characterize variability is critical, as it is to optimize model performance according to the population where the model will be used. Therefore, variability-resilient AI training should apply both at the initial dataset learning and during its prospective production cycle, as described in this paper.

Similarly, ML responds suboptimally at overlapping information zones, that is, where repeated similar cases show distinct outcomes, bounding the Bayes error, the minimum and irreducible error rate achievable by a model. RAI should help delineate these zones during training, enabling reviewing, skipping, or fixing these cases or suggesting complementary models trying to optimize prediction at these zones.

RAI training should account for user requirements regarding the expected performance, including cost-benefits for each miss-correct classification, targets for sensitivity, specificity, or positive predictive value, and their trade-offs. These estimations are key in unbalanced outcomes and against FRA-unbalanced subpopulations. Lastly, training should enable prospective explainability and interpretability in the prediction phase.

### Prediction

Directly related to clinical decision-making, resilience becomes critical in prediction. Balancing clinical usefulness with time-efficiency—with minimum intrusiveness—RAI should encompass fully automated support with smart human-AI interaction: a fully automated RAI will act without additional user feedback, while a semiautomated RAI will help improve or investigate the results through additional feedback.

Determinant for the user’s adherence to the AI decision processes, RAI predictions should be explainable and interpretable [23,24]. Interpretability means understanding in human terms why a model provides an output and how it could vary from changes in the input—for example, through a sensitivity analysis. Explainability means understanding the internal mechanics of a model driving causality in a specific decision-making process.

Uncertainty is intrinsic to decision-making. Therefore, informing and quantifying uncertainty in health AI would significantly increase the system’s trustworthiness and confidence in derived decisions. Particularly relevant are input cases showing unprecedented data patterns. RAI outcomes should count, where relevant, with CIs or levels. Likewise, RAI should handle potential input issues by identifying DQ-affected inputs, smartly encoding missing data, and identifying lacking optional information potentially relevant to the prediction.

Toward CDSS transportability, endurance, and fairness, RAI should enable the comparison of predictions obtained at multisite consensus or local level, identify potential changes in the outcome over time, or check for differences at distinct FRA-related subpopulations. Of note is that RAI should compare our case with similar past cases with validated outcomes, particularly beneficial in rare cases, and warn about potential FRA violations.

### Production

Once an AI is in routine clinical use, changes can occur in the data acquisition contexts, the target populations of the model, or in clinical knowledge and user requirements. The dataset shifts described before in the training block can still occur in production, either over time or using the AI in different settings from those we initially trained it in. To avoid unexpected biases and obsolescence, we must continuously benchmark AI performance on target populations and enable automated or semiautomated self-adaptation mechanisms to these changes.

Besides benchmarking model performance in clinical indicators—for example, number of hospital readmissions in readmission prediction—or confusion matrix-derived metrics—for example, sensitivity and specificity—an RAI in production should monitor dataset shifts, user-defined DQ rules, user requirements—for example, deprecated or new International Classificafion of Diseases codes—and continuously benchmarking FRA.

On any significant change in AI performance, or even in advance of them, RAI should be able to self-adapt its knowledge over time or when transporting a model to other settings. Further, changes in requirements and clinical knowledge should be easily incorporated into previously built models without requiring a whole reengineering process.

### Regulation

Production health AI and CDSS require regulations for medical device products, such as the European Union (EU) Medical Device Regulation [25], and specific regulations for AI, including the European Regulatory Framework for AI or AI Act [8,9,11]. The US Food and Drug Administration provides the AI and ML in Medical Devices white paper and its AI and ML Software as a Medical Device Action Plan [26,27].

The flexibility of current regulations on putting trustworthiness and patient safety as the top priority accommodates most of the described resilient AI needs. However, the wide RAI casuistry, involving more system self-decisions and higher levels of interaction and data access, might deserve more detailed definitions.

To promote AI while addressing potential risks, the EU AI Act includes a list of prohibited AI practices, rules for high-risk AI systems, transparency obligations, liability rules, and an innovation support legal framework, all consistent with the EU General Data Protection Regulation (GDPR). Specifically, the EU AI Act Article 15—Accuracy, Robustness, and Cybersecurity—Section 4 states that high-risk AI systems shall be as resilient as possible regarding errors, faults, or inconsistencies that may occur within the system or the environment in which the system operates, in particular, due to their interaction with natural persons or other systems. How to implement these resilience features is left to the system designers. At the simplest level, RAI could stop or warn users of unexpected situations, such as potential uncertainties or FRA risks at outputs or inputs. How this information is presented and interacted with, since it potentially affects decision-making, might be the subject of more specific regulations. Further, Article 15, Section 5, also claims resilience against attempts to exploit system vulnerabilities.

However, in addition to during system operation, issues can occur as well at initial model training and in prospective retraining, where alternative resilience features could also take place. In this regard, Article 10—data and data governance—claims that data governance and management practices can be used to mitigate these data-related biases. However, fully resilient AI should, to some extent, make self-decisions for automated data curation or FAIRification procedures, which could be applied both during training and system operation. These may require stricter regulation and a justification supported by the added value of this process.

An innovative regulatory aspect for RAI focuses on self-adaptive AI, which automatically updates its knowledge to maintain performance and avoid obsolescence during production or, similarly, when transporting the AI to other settings. RAI self-updates should avoid, to some extent, new conformance evaluations. Self-adaptive RAI might also need data access regulations for benchmarking and retraining AI while respecting data security and privacy, such as in compliance with the EU GDPR [28]. Besides, accessing the EHRs for similar cases search and comparison, and the need for sensitive, FRA-related information potentially improving the system performance should also be regulated.

Lastly, for black-box models significantly outperforming other interpretable solutions, regulations should consider mitigation through resilient explainable and FRA features rather than limiting the AI use indications [29,30].

## Available Methods and Solutions

### Overview

Next, we describe a collection of currently available methods and solutions with a feasible while relevant use to address to some extent the RAI objectives and resiliency functions defined in the previous section.

### Data Quality

The assurance of DQ can apply to the whole health data and AI lifecycles. Initially, AI is typically trained on secondary use data, of which quality and utility for AI should be labeled appropriately for trustworthy use. The current proposal for a regulation of the EHDS addresses the necessity of labeling the DQ and utility (Article 56) for data uses including research and personalized medicine, as is the case for AI.

DQ assurance can be specified based on DQ dimensions, which characterize data attributes facilitating or impeding its expected use. Dimension definitions vary according to the field of study [17,18,31,32]. Some dimensions are intrinsic to the data contents, including completeness, correctness, plausibility, or stability. Others relate to data access, including availability, accessibility, or security. Since potentially hindering or impeding AI development and use, we set intrinsic and access dimensions as the initial DQ targets in RAI.

Addressing DQ generally involves costly processes, from quality rules checks and exploratory analysis to data curation. Simple rules can find missing data footprints—blank spaces or, in the worst case, negative numbers such as “–9.” Logic rules defined by experts can help find implausible patterns in and between variables. Principal component analysis plots can help find apparently plausible but unlikely data, such as outliers. Further, information variability methods can help delineate temporal and source variability [22,33,34].

Addressing faulty data in training includes skipping faulty cases or recovering faulty values through data imputation methods. DQ flags can occasionally provide information relevant to AI performance, such as in the missing not-at-random case described in section 2. Further, as described in this paper, continual learning and model transportability methods can help address data variability in AI training.

Besides, DQ issues for AI can also occur when data is inputted into the AI systems, either being created de novo or passed through the EHR. At this stage, DQ handling methods can warn or automatically update missing, implausible, or outlying inputs with the most likely values given a context. However, any fully automated modification should be notified, and its effect on the output quantified through sensitivity analysis. This can be achieved through smart human-computer interaction and explainable AI methods.

### Uncertainty Management

In health AI, data-related uncertainty can stem from epistemic or aleatoric factors, including lacking or faulty data or from pure statistical randomness in data or information overlap [35-37].

Quantification of uncertainty in AI focuses mainly on prediction accuracy, that is, informing about variability and confidence of the outputs. Available approaches include deterministic, Bayesian, ensemble, and augmentation methods [35,37]. Deterministic methods aim to predict jointly an output and its variability in single models, thus requiring this information at training. Bayesian methods model the statistical distribution of AI parameters—for example, the coefficients of logistic regression—rather than as single values, capturing the training data variability and enabling an output distribution from which to estimate a mean and CI. Bayesian methods include Monte Carlo dropout or Gaussian mixture models. Ensemble methods estimate multiple models—for example, through boosting or bagging approaches—in which combined outputs conform to a distribution. Lastly, augmentation methods apply artificial but realistic perturbations to inputs enabling multiple predictions which then also conform to a distribution.

Further, uncertainty from lacking information is particularly relevant in open inputs, including free text and dialogue systems such as in large language models (LLMs). It can be managed through deep learning network architectures, such as using recurrent neural networks that treat the input as a sequence with order information, recursive neural networks that treat the input as a hierarchical structure, transformers masked models such as BERT, where the different parts of an input sequence can influence each other through attention mechanisms [38], or using input embedding layers, where partial inputs can be expressed as indexes of dense vector representations [39].

### Continual Learning and Model Transportability

Continual learning provides AI with mechanisms to self-adapt to information changes over time [40]. The model knowledge, generally represented as hyperparameters—for example, an artificial neural network layout—and parameters—the network weights—updates as new data batches arrive. Continual learning must balance forward and backward knowledge transfer, that is, rapidly adapting to dataset shifts while avoiding a catastrophic forgetting of important predictive past patterns. When using an AI model at settings or sites different from where it was trained, we face the problem of model transportability.

The continual learning and model transportability fields encompass similar methods from diverse ML paradigms, including online, active, transfer, or multitask learning [41]. Given their adaptative flexibility, these methods generally relate to artificial neural networks and deep learning. However, other simpler methods, including logistic regression or random forests, can also use batch or iterative learning. Of note is that these methods can update their knowledge without needing past data, conforming to data access regulations. Continual learning and model transportability can also benefit from infrastructure and organizational practices, including interoperability and domain knowledge management. Further, recent ML methodologies such as ML operations consider continual benchmarking and model updating [42]. Of note is that the use of AI foundation models, pretrained across various domains, can be of potential benefit to optimize resilience in continual learning and model transportability [43].

### Fundamental Rights Assurance

Health AI involves potential risks for FRA regarding equality and nondiscrimination, economic and social rights, access to a fair trial and effective remedies, protection of personal data, or good administration [10]. RAI can address some of these rights by attaching to principles including the EU Charter of Fundamental Rights [44], the Ethics Guidelines for Trustworthy AI [45], or the GDPR [28]. In fact, FRA is at the core of medical AI regulations [46]. A successful implementation demands specific computational methods handling potential algorithmic biases.

We can classify FRA computational methods into pretraining, in-training, and posttraining methods [47,48]. Initially, we should identify available or derived variables potentially defining FRA-sensitive subpopulations, such as gender, race, or socioeconomic status. In pretraining methods, we compensate underrepresented subpopulations or imbalanced outcomes via resampling methods. Reweighting mechanisms can mitigate discrimination in outcomes. Further, we can remove or obfuscate FRA-sensitive variables if they do not affect model performance or usability. Besides, in-training methods focus mainly on defining objective functions or constraints in the AI learning process to compensate for unfair performance metrics in sensitive subpopulations—for example, optimizing the false-negative rate for a discriminated subpopulation. In screening and classification tasks, we can set subpopulation-specific cost benefits in confusion matrices or use specific deep learning loss functions—that is, the functions that measure the difference between the model prediction to the true answers to learn the network weights. Lastly, posttraining methods adapt a model’s outcome to the sensitive situations of the tested individual—for example, setting custom receiver operating characteristic curves and decision thresholds or readjusting uncertain outcomes to favor sensitive subpopulations.

DQ and variability methods can also help detect unfair differences in data representations, DQ levels, or model performance across subpopulations. Another FRA aspect is decision-making transparency, where the models’ interpretability is highly important.

### Human-Computer Interaction

Resilient human-computer interaction is key to trustworthy AI and FRA. The methodological drivers for RAI by human-computer interaction include user experience and interface design methods [49], toward developing resilient user-centered CDSSs from the beginning [50], and AI and CDSS engineering aiming for dynamic and explainable AI [23,24].

Dynamic human-computer interaction in AI can use sequential prediction models that generate and update outcomes on partial inputs as new information is introduced, such as in recurrent or transformer neural networks [24]. Sequential models can establish a smart human-computer dialogue, particularly useful for sequential anamnesis inputs and natural language processing, such as using LLM.

Regarding explainable AI, the simpler the model, the more interpretable it is. For example, we can easily reason about a logistic regression behavior based on the feature’s coefficients. Explainable AI post hoc methods provide explanations for less interpretable or “black box” models, which typically perform higher in complex tasks, including natural language processing or medical imaging. Post hoc local methods explain specific predictions, including the model-agnostic Shapley values or neural network gradient attribution techniques such as saliency maps. Post hoc global methods estimate each feature’s average importance for any prediction. Surrogate interpretable models in points close to the test case can assist in explaining individual predictions from less-interpretable models [51]. Lastly, explainable AI local techniques can provide a sensitivity analysis for the outcomes.

### Regulation

Current regulations supporting the development and maintenance of RAI solutions AI include those described in section 2, namely the EU AI Act, the EU Medical Device Regulation, the AI Action Plans by the Food and Drug Administration, and the proposal for the EHDS.

As a first step to facilitate the transportability of production AI systems, the EHDS provides a set of common specifications (Article 23) and a list of interoperability and security requirements (Annex II). These include structural and semantical requirements, and requirements related to DQ, patient safety, and data protection.

Some of the harmonized rules (HRs) that provide concrete details on how to meet the EU AI Act’s goals align with our aim for RAI in health: high DQ and proper statistical properties in training, testing, and validation are essential for the performance of AI and to avoid biases—HR-67; explainability and transparency to avoid opacity is key to use the system appropriately—HR-72; the system performance should be consistent throughout its lifecycle and monitored in real-life settings—HR-74; and the systems should be resilient against errors, faults, inconsistencies, or unexpected situations, as well as malicious actions, to avoid erroneous, potentially harmful decisions or biased outputs—HR-75.

Regarding self-adaptive AI, once a system is put into service, the EU regulatory framework for AI suggests providing rules establishing that changes in the algorithm and its performance, predetermined by the provider and assessed at conformity assessment, should not constitute a substantial modification—HR-128. Similarly, the American Medical Informatics Association suggests a set of recommendations for the safe, effective use of self-adaptive CDSS and their prospective regulation concerning: the design and development based on transparency metrics; implementation using standards for communication and planning retraining criteria; in-situ evaluations and testing; and on-going monitoring including system maintenance and user training [52].

### Others

Further transversal approaches can benefit RAI. Federated learning provides algorithms and interoperability infrastructure for multisite AI learning, enabling secure model transportability and continual learning addressing potential data access regulations. In the prediction phase, fast nearest-neighbor search and phenotyping algorithms can proactively display relevant comparative past cases [53-55]. Lastly, we bring attention to the proposed commandments of ethical medical AI by Muller et al [30], which can help design RAI-enabled CDSS while encompassing human-AI decision roles.

**Figure 3:**
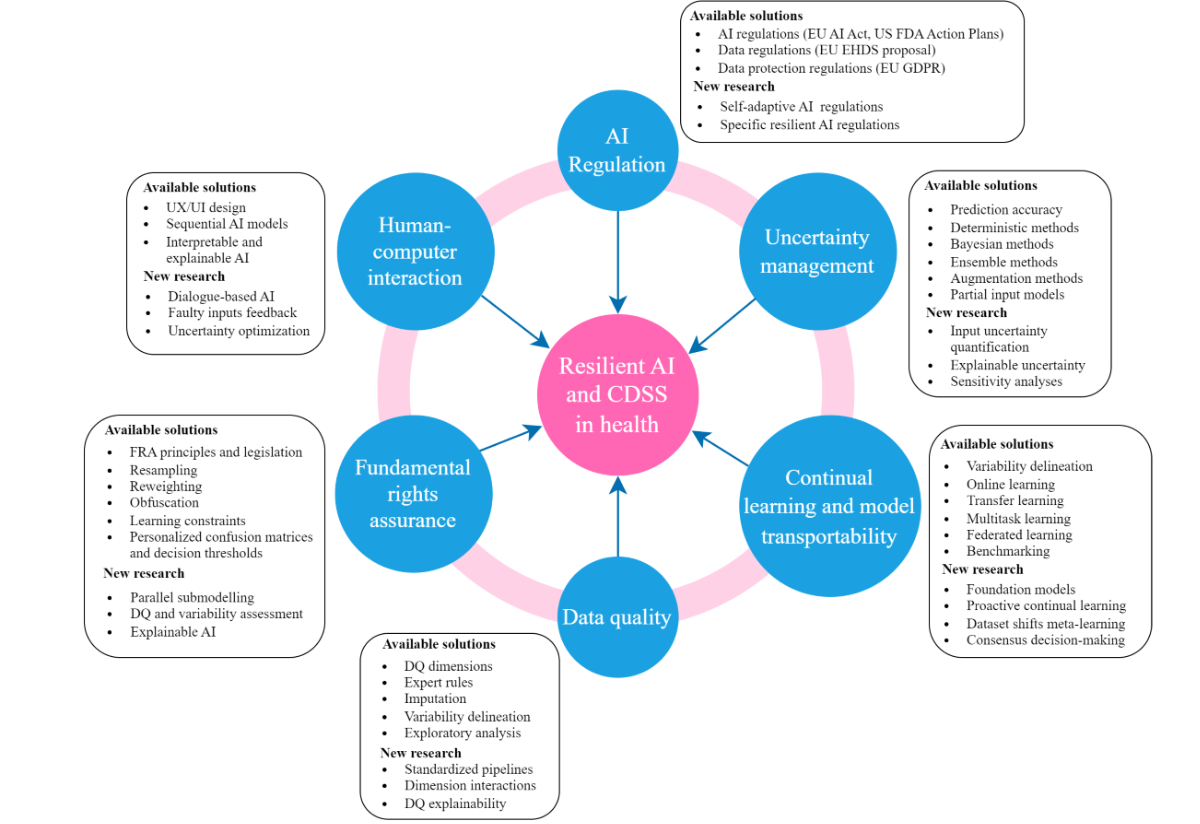
Summary of available solutions and new proposed research for resilient AI in health and CDSS. AI: artificial intelligence; CDSS: clinical decision support systems; DQ: data quality; EHDS: European Health Data Space; EU: European Union; FDA: Food and Drug Administration; GDPR: General Data Protection Regulation; UX/UI: user experience and interface.

Figure 3 summarizes the main solutions described in this section, linking to potential new research aims, as described in this paper in more detail.

## Research Agenda

Putting the methods and solutions described into practice would be already a huge step toward RAI and trustworthy CDSS (see Textbox 2 for an example of a desired use of an RAI-enabled CDSS). However, this is not free of challenges. The development complexity and costs can be considerable, regulations must be met or extended, and additional validation of the methods is needed. Fortunately, user acceptance and organizational culture regarding RAI may benefit from conventional AI, given its increased, user-centered flexibility. Next, we propose several research aims to address current and future challenges in RAI.

**Example desired use of a resilient artificial intelligence–enabled clinical decision support system following the example from emergency medical dispatch in Textbox 1.**

**Context**

- Suppose a European emergency medical dispatch center develops a clinical decision support system based on resilient artificial intelligence (RAI) for triage support. The aim is to predict the life-threatening situation of incidents based on the live transcription of the during-call conversations to support resource allocation.

**Training**

- The initial RAI learning is fed from a raw database containing transcriptions, in situ maneuvers and diagnoses, transport, and hospitalization—as the final gold standard—data of the last 10 years. The system vendor and its triage tree changed during this period, leading to different conversation types. Further, the International Classification of Diseases codes at hospitalization were updated from version 9 to version 10. The RAI training is based on a continual, deep learning methodology; the neural network is learnt in temporal batches through a continual fine-tuning strategy. Therefore, the final model will retain past knowledge, but recent patterns will be more relevant. The training also relied on resiliency functions that permitted, for example, alerting about outlying operators with training data biased toward overclassification, prospective uncertainty quantification of inputs based on a Monte Carlo dropout function, and suggesting a parallel model for female cases with poor information in cardiac disease incidents with positive outcome to avoid false negatives.

**Prediction**

- The case from Textbox 1 is inputted into the system, which classifies it as a high-uncertainty case using the uncertainty quantification function. The system automatically asks for potentially relevant information to reduce uncertainty, such as the use of drugs or recent anxiety attack episodes. In light of neither changes in the input nor interoperability with the patient EHR to retrieve this data, the system uses a reject option-based classification function to classify the case as life-threatening to avoid potentially biased decisions.

**Production**

- A continuous artificial intelligence (AI) benchmarking system is used, counting with secure interoperability with gold-standard feedback data and automated inspection of causes of changes. The system eventually detects reduced performance in older adults and newborns, finding multidrug-resistant bacterial infections associated with these cases, and suggests retraining the model with these new data.

**Regulation**

- The system is a high-risk AI system in compliance with the European Union (EU) AI Act, ensuring its safe and trustworthy use. It conforms with the European Health Data Space proposal’s common specifications, interoperability, and security requirements. An updated regulation framework considered the digital document with clinically explained motivation for the retraining described before as a valid source for self-conformance of the system to continue its use after its self-update.

We must standardize pipelines for fully automated and semiautomated DQ processing during training. The implications of DQ dimensions interactions, for example, across-site missingness patterns, should be studied. Excessive curation might lead to an unreal dataset; therefore, combining curation with using affected informative patterns—for example, informative missingness [56]—should be further studied. In prediction, we must investigate user-centered human-computer interaction approaches against faulty inputs, for example, using automated DQ corrections and DQ-related sensitivity analysis. Legal aspects for data curation, especially if automated, should also be realized technically [57].

Uncertainty quantification can benefit from addressing input uncertainty besides outcome uncertainty. Input uncertainty quantification could combine imputation and data augmentation methods. This approach can also help analyze the effect of DQ on output uncertainty and lead to input-outcome sensitivity analysis. Additionally, explainability might improve from uncertainty quantification at specific decision stages, for example, visualizing uncertainty at neural network branches.

Conversational, dialogue-based RAI interactions, smartly asking for new parameters anticipating the user needs, while minimizing input costs following an uncertainty-reduction targeted dialogue, are key research aims. This could be achieved by combining recurrent AI with dynamic human-computer interaction, for example, through voice or text-based LLMs [58,59], enabling AI to work with partial information, the natural case in medicine. Besides, user-centered design with a focus on explainability deserves specific research toward its acceptance [60].

Human-AI interaction is key to FRA since it influences users’ decision-making. Previous methods for uncertainty quantification, DQ assessment, or RAI interactions shall be put in common with AI and health data regulations, for example, through specific HRs and methods enabling FRA as a priority layer in RAI-based CDSS interactions.

The use of foundation models in CDSS development should be further validated for safe and trustworthy clinical use. They can enable rapid development and model transportability, and show promising behavior to fill knowledge gaps in the training data, increasing generalization [15,16,43]. However, risks and uncertainty must still be thoroughly quantified. Besides, foundation models will still need to be updated as data changes through continual learning methods.

Continual learning can evolve from reactive to proactive. Current resilience against dataset shifts is constrained by the need for new labeled data to update the model’s parameters, which is always one step behind the actual context. Further than adapting periodically or after significant shifts, proactive continual learning can aim to anticipate changes. Domain adaptation methods—for example, transductive learning—can help update knowledge from cases before their gold-standard outcomes are available [61,62]. Besides, comprehensive variability characterizations in historical datasets can provide an extensive knowledge base of variability patterns, potentially enabling the forecasting of changes [22,63]. Overall, proper benchmarking for continual learning in health is still challenging, where we motivate the availability of publicly available patient-level datasets including the cases’ date of acquisition.

Predictions based on the “consensus” of parallel models may support human decision-making resiliency—not generally based on a single mental process—through cooperative knowledge from different perspectives. Parallel models can apply to FRA-sensitive subpopulations, missingness patterns, multisite and temporal variability, and high uncertainty, overlapping-information zones. However, this can risk the CDSS user understanding; for example, what is the trustworthiness balance between local and multisite outcomes? Transparent, distributed, and continuous AI benchmarking is key. Complementarily, consensus decision-making can benefit from research in context and logic-based argumentation algorithms [64].

Specific resilience and self-adaptiveness features must be included in health AI regulations. The adaptability of AI to different settings and the temporal evolution of medical practice are recognized regulatory challenges [52,65]. Any change in an AI production system currently requires human intervention and conformance evaluation. Therefore, automatically providing a clinically explainable justification for why and how self-adaptive AI is updated without human intervention—besides planned retraining—is significant future work. Further, we must standardize AI documentation and record-keeping practices to define the rules firing a self-adaptation process and keep track of the latter—for example, based on performance changes or dataset shifts—and, similarly, justify automated DQ curation processes both at training and prediction.

Self-adaptiveness and federated learning infrastructures should be validated and regulated for CDSSs’ retraining, production use, and benchmarking with data access limitations. In cases when protected information is required—for example, for training or similarity searches—synthetic data fabrication can be a solution.

Lastly, future work in RAI can address unsupervised and reinforcement learning. Unsupervised learning aims to discover natural subgroups in data. Generally applied at the population level—for example, to find potential immune response patterns in blood tests—it can also apply at the patient level—for example, for tissue segmentation in medical imaging. Besides, reinforcement learning aims to learn optimal decision-making choices in nonstationary environments—for example, to optimize intensive care unit procedures [66]. Improving the resilience of both will potentially improve their contribution to actual clinical practice.

## Conclusions

Resilience is a significant factor in the success of health AI. Health data is imperfect, incomplete, and permeated with variable representations. However, this is not wrong. Instead of artificially modifying data for AI, AI should be resilient to the data nature. Methods enabling RAI involve disciplines including DQ, uncertainty management, continual learning, model transportability, foundation models, conversational AI, human-computer interaction, and regulatory aspects. Their implementation and specific research in RAI will define new-generation CDSSs and maximize trustworthiness in AI-enabled health care.

## Appendix

Multimedia Appendix 1 Potential data-related uncertainties, variability, and biases for health artificial intelligence at the training and prediction phases.

## Abbreviations

AI: artificial intelligence
CDSS: clinical decision support system
DQ: data quality
EHDS: European Health Data Space
EHR: electronic health record
EU: European Union
FRA: fundamental rights assurance
GDPR: General Data Protection Regulation
HR: harmonized rule
LLM: large language model
ML: machine learning
RAI: resilient artificial intelligence
RWD: real-world data
