# Supplementary material for: Resilient Artificial Intelligence in Health: Synthesis and Research Agenda Toward Next-Generation Trustworthy Clinical Decision Support
Source: J Med Internet Res. 2024 Jun 28;26:e50295. doi: 10.2196/50295 (PMC11245653; doi:10.2196/50295)
Supplement: Multimedia Appendix 1 [file jmir_v26i1e50295_app1.pdf]

Resilient Artificial Intelligence in Health: Synthesis and Research Agenda Toward Next-Generation Trustworthy Clinical Decision Support

Carlos Sáez[1] (carsaesi@upv.es), Pablo Ferri-Borredà[1], Juan M García-Gómez[1]

[1]Biomedical Data Science Lab (BDSLab), Instituto Universitario de Tecnologías de la Información y Comunicaciones (ITACA), Universitat Politècnica de València (UPV), Camino de Vera s/n, Valencia 46022, España

Table S1 *Potential biases for AI in health that can be attributed to data. To help target the design of resilient health AI approaches we categorize the sources of the problem in individual and sample data levels, and associate them to the two phases of supervised machine learning, namely training (e.g., estimating the optimal parameters of a neural network for 30-day mortality prediction) and prediction (e.g., applying the neural network to estimate the 30-day mortality risk of a new, unobserved case).For simplicity, we assume the use of consistently formatted data with a priori known variables and values, as defined in a data dictionary or health information standard, and, therefore, with a minimum level of syntactic and semantic interoperability if integrating data from multiple sites.*

Problem \ AI phase		Training	Prediction
Individual level	Missing data<br>• Unknown values for required*[1] variables	Cannot include directly individuals with missing data in the model	Cannot apply the model
	Unavailable information<br>• Lacking information at inputs of varying size<br>• Unknown values for optional*[1] inputs<br>• Potentially relevant variables not included	It can cause dimensionality problems due to data sparsity, what can hinder or even impede the learning process<br>The models may not achieve a sufficient performance for the task	The prediction shows higher uncertainty, e.g. increasing the confidence interval for the prediction
	Incorrect data<br>• Untrue recorded information, but apparently plausible<br>• Repeated patterns at sample level can lead to information overlap	Random untrue data can lead to models with higher variance<br>Repeated patterns in untrue data can lead to biased models from a wrongly represented population	The prediction provides biased, untrue results
	Implausible data<br>• Implausible variable values or combination of values, which can be based on some given context<br>• Outlier cases – although in cases these can be likely	The learned model is biased and/or with more variance	The prediction provides biased results through out of sample/range extrapolation
Sample level *[4]	Information overlap<br>• Distinct information representations for equivalent real-world situations, beyond natural statistical variance	The learned model is biased and/or with more variance	The prediction shows higher uncertainty, e.g. increasing the confidence interval for the prediction
	Unrepresented or underrepresented subpopulation<br>• A target population for the model is not included or not as frequent as it is in reality*2	The learned model is biased and/or untrustworthy in those subpopulations	The prediction provides biased results for inputs of those subpopulations, potentially against fundamental rights assurance
	Temporal variability<br>• The statistical frequencies of variables shift over time, either individually, jointly or conditionally *3<br>• Variables or values get deprecated or new ones are introduced	The model is forced to learn multiple patterns in the conditional probabilities of data occurring over time, some of them not to be observed in the future, thus diminishing its performance for new cases, increasing model obsolescence	The prediction provides biased results for new, unobserved cases which are more similar to latest patterns, or from new patterns not present yet in training
	Source variability<br>• The statistical distributions of variables shift across data generating sources (sites, persons, etc.)<br>• Some variables or values are not available across sites providing data to an integrated repository	The model is forced to learn multiple patterns in the conditional probabilities of data occurring across sources, where the performance may vary if using global vs local models	The prediction provides biased results both at sources with isolated patterns and at sources with more general patterns due to the model fit to each other

*[1] By default in traditional structured data all the input variables are required in a model, however, in cases such as text or dialogue based or AI, the inputs can be of varying size, leading to potentially sparse input representations.
*[2] Real imbalance should not be considered, such as the natural less prevalence of a target disease class in classification problems.
*[3] This includes gradual, abrupt and seasonal shifts.
*[4] Repeated individual level problems in multiple instance can lead to merged types of problems, i.e. source variability due to different missingness patterns.
